# Mesolimbic opioid-dopamine interaction is disrupted in obesity but recovered by weight loss following bariatric surgery

**DOI:** 10.1038/s41398-021-01370-2

**Published:** 2021-05-01

**Authors:** Henry K. Karlsson, Lauri Tuominen, Semi Helin, Paulina Salminen, Pirjo Nuutila, Lauri Nummenmaa

**Affiliations:** 1 Turku PET Centre, Turku University Hospital and University of Turku, Turku, Finland; 2 Institute of Mental Health Research, University of Ottawa, Ottawa, ON Canada; 3 Department of Surgery, University of Turku, Turku, Finland; 4 Division of Digestive Surgery and Urology, Turku University Hospital, Turku, Finland; 5 Department of Endocrinology, Turku University Hospital, Turku, Finland; 6 Department of Psychology, University of Turku, Turku, Finland

**Keywords:** Molecular neuroscience, Neuroscience

## Abstract

Obesity is a growing burden to health and the economy worldwide. Obesity is associated with central µ-opioid receptor (MOR) downregulation and disruption of the interaction between MOR and dopamine D_2_ receptor (D_2_R) system in the ventral striatum. Weight loss recovers MOR function, but it remains unknown whether it also recovers aberrant opioid-dopamine interaction. Here we addressed this issue by studying 20 healthy non-obese and 25 morbidly obese women (mean BMI 41) eligible for bariatric surgery. Brain MOR and D_2_R availability were measured using positron emission tomography (PET) with [^11^C]carfentanil and [^11^C]raclopride, respectively. Either Roux-en-Y gastric bypass or sleeve gastrectomy was performed on obese subjects according to standard clinical treatment. 21 obese subjects participated in the postoperative PET scanning six months after bariatric surgery. In the control subjects, MOR and D_2_R availabilities were associated in the ventral striatum (*r* = .62) and dorsal caudate (*r* = .61). Preoperatively, the obese subjects had disrupted association in the ventral striatum (*r* = .12) but the unaltered association in dorsal caudate (*r* = .43). The association between MOR and D_2_R availabilities in the ventral striatum was recovered (*r* = .62) among obese subjects following the surgery-induced weight loss. Bariatric surgery and concomitant weight loss recover the interaction between MOR and D_2_R in the ventral striatum in the morbidly obese. Consequently, the dysfunctional opioid-dopamine interaction in the ventral striatum is likely associated with an obese phenotype and may mediate excessive energy uptake. Striatal opioid-dopamine interaction provides a feasible target for pharmacological and behavioral interventions for treating obesity.

## Introduction

The prevalence of obesity is dramatically increasing and there is an urgent need for novel efficient therapies. Numerous studies point towards the role of the brain in the development and maintenance of obesity^1,2^. Previous studies indicate that both opioid and dopamine systems in the brain’s reward circuit are dysfunctional in obesity. The endogenous opioid system has been linked to hedonic aspects of feeding in animals^3,4^. In humans, both µ-opioid receptor (MOR) antagonists and inverse agonists have been shown to reduce eating behavior^5,6^. Previously, decreased MOR availability has been observed in the reward circuit among obese subjects^7,8^. Thus, aberrant opioid functioning in obesity may diminish the opioid-dependent rewarding effects of eating. Alterations in dopamine D_2_ receptor (D_2_R) expression and function in obesity have been observed in some^9–12^ but not all human imaging studies^8,13^. It is possible that the relationship between D_2_R availability and measures of obesity is not linear, but quadratic^14^ or age-dependent^15^. Alternatively, it is possible that the effects of obesity on D2R are mediated via the MOR system.

Tight interaction between dopaminergic and opioidergic systems has been proposed to underlie human reward functions^16^, but only a few studies have actually investigated this issue. In humans, dopamine-releasing drugs such as cocaine and amphetamine lead to endogenous opioid release^17–19^. In rats, both D_2_Rs and MORs are closely connected in the striatum, which can be morphologically divided into striosome/patch and matrix compartments. MORs can control the release of dopamine by inhibiting GABAergic interneurons in VTA^20–25^. Furthermore, VTA dopamine neurons express MOR postsynaptically, and direct inhibition between MOR and dopamine neurons exists without GABAergic signaling^26^.

Cross-talk between opioidergic and dopaminergic systems may underlie aberrant reward-related behaviors, such as excessive feeding. In rats, intravenous administration of MOR agonists triggers dopamine release and feeding^27^, while MOR antagonists block dopamine release and reduce food consumption^28^. Finally, in vivo PET data from humans show that there is a close interaction between MOR and D_2_R receptors in the reward circuit among non-obese subjects, while this interaction is disrupted in the ventral striatum among obese subjects, potentially contributing to obesity^29^. However, it remains unclear whether the dysfunctional MOR/D_2_R interaction reflects a vulnerability endophenotype for obesity, or whether it develops as a consequence of the obese state.

Bariatric surgery is the most effective method for weight loss in obesity^30^. The surgical procedure significantly lowers appetite^31^, but the actual molecular brain mechanisms behind this are still poorly understood. Bariatric surgery provides a powerful method for investigating changes in neuroreceptor systems and opioid-dopamine interaction after weight gain. Previous studies have investigated the effects of bariatric surgery and following weight loss to separate receptor systems, showing mainly unaltered D_2_R availability and normalized MOR availability^32–35^. Here we tested whether bariatric surgery-induced weight loss could recover the dysfunctional opioid-dopamine interaction in the obese.

## Subjects and methods

The study was conducted in accordance with the Declaration of Helsinki and approved by the Ethical Committee of the Hospital District of South-Western Finland (SleevePET2, NCT01373892, http://www.clinicaltrials.gov). All participants gave a signed informed consent form prior to scans.

### Subjects

We recruited 25 morbidly obese women (mean BMI 41 kg/m^2^) eligible for bariatric surgery. Either Roux-en-Y gastric bypass or sleeve gastrectomy was performed as their standard clinical treatment. Four subjects discontinued the study for personal reasons, and 21 subjects participated in the postoperative scanning six months after the surgical procedure. 20 non-obese healthy women (mean BMI 22 kg/m^2^) formed the control group. Data for this patient cohort have been reported previously^8,29,34^. The sample size was determined by a priori power analysis based on our previous studies^34^. Characteristics of the subjects are presented in Table 1. Clinical screening of the subjects included history, physical examination, anthropometric measurements, and laboratory tests. Exclusion criteria involved opiate drug use, binge-eating disorders, neurological and severe mental disorders, substance abuse, excessive alcohol consumption (more than eight units per week) determined by clinical interviews, medical history, and blood tests. None of the controls smoked tobacco, but 8 obese subjects were smokers (3–15 cigarettes per day). Antidiabetic, antihypertensive, and cholesterol-lowering drugs were paused prior to the study.

**Table 1:** Characteristics of the subjects.

	Obese preoperative (*N* = 25)	Obese postoperative (*N* = 21)	Healthy control subjects (*N* = 20)
Age (y)	41.2 ± 9.2	–	42.0 ± 13.2
BMI (kg/m^2^)	41.3 ± 4.1	31.9 ± 4.4	22.4 ± 2.6
Percentage of fat (%)	50.3 ± 6.7	43.2 ± 4.2	30.6 ± 6.4
Tobacco smokers/non-smokers (*N*)	8/17	5/16	0/20
Amount of alcohol use (units per week)	1.7 ± 1.8	N /A	2.9 ± 2.3
Injected activity of [^11^C]carfentanil (MBq)	253.2 ± 11.6	252.1 ± 15.0	251.2 ± 8.4
Injected activity of [^11^C]raclopride (MBq)	247.9 ± 20.8	254.5 ± 10.9	258.3 ± 15.7

Data are presented as mean ± SD.

### Image acquisition and quantification of receptor availability

We measured D_2_ receptor availability with the antagonist [^11^C]raclopride^36^ and µ-opioid receptor availability with the high-affinity agonist [^11^C]carfentanil^37^ using positron emission tomography (PET) on two separate visits. Subjects were scanned again with both radiotracers six months after bariatric surgery. Radiotracer production has been described previously^8^. Both radioligands had high radiochemical purity (>99%). Before scanning, a catheter was placed in the subject’s left antecubital vein for tracer administration. The Head was strapped to the scanner table in order to prevent head movement. Subjects fasted two hours prior to scanning. A CT scan was performed to serve as an attenuation map. The clinical well-being of subjects was monitored during the scanning.

We injected both tracers as a bolus in separate scans on separate days. Injected amounts of [^11^C]carfentanil and [^11^C]raclopride are presented in Table 1. After injection, radioactivity in the brain was measured with the GE Healthcare Discovery^TM^ 690 PET/CT scanner (General Electric Medical Systems, Milwaukee, WI, USA) for 51 min, using 13-time frames. MR imaging was performed with Philips Gyroscan Intera 1.5T CV Nova Dual scanner to exclude structural abnormalities and to provide anatomical reference images for the PET scans. Anatomical images (1 mm^3^ voxel size) were acquired using a T1-weighted sequence (TR 25 ms, TE 4.6 ms, flip angle 30°, scan time 376 s).

All alignment and coregistration steps were performed using SPM8 software (www.fil.ion.ucl.ac.uk/spm/) running on Matlab R2012a (The Mathworks Inc., Sherborn, Massachusetts). To correct for head motion, dynamic PET images were first realigned frame-to-frame. The individual T1-weighted MR images were coregistered to the summation images calculated from the realigned frames. Regions of interest (ROIs) for reference regions were drawn manually on MRI images using PMOD 3.4 software (PMOD Technologies Ltd., Zurich, Switzerland). The occipital cortex was used as the reference region for [^11^C]carfentanil and cerebellum for [^11^C]raclopride. Receptor availability was expressed in terms of *BP*_ND_, which is the ratio of specific to non-displaceable binding in the brain. *BP*_ND_ was calculated by applying the basis function method for each voxel using the simplified reference tissue model (SRTM) with reference tissue time-activity curves (TAC) as input data^38^.

The subject-wise parametric *BP*_ND_ images were normalized to the MNI space using the T1-weighted MR images and smoothed with a Gaussian kernel of 8 mm FWHM. Anatomic regions of interest were generated in the ventral striatum, dorsal caudate nucleus, and putamen using the AAL^39^ and Anatomy^40^ toolboxes. Statistical analysis was performed as described earlier^29^. In the ROI analysis, Pearson correlation was calculated between the tracer-wise *BP*_ND_s in the striatal regions of interest. Fisher’s *z*-test was used for quantifying whether ROI-level Pearson correlations between the [^11^C]raclopride and [^11^C]carfentanil *BP*_ND_ values were statistically different between groups. Normality assumption was tested with the Kolmogorov-Smirnov test.

To determine the striatal volumes in all three groups, we used T1 images and automated FreeSurfer volumetric analysis (Version 7). Preoperative differences in striatal volumes between obese and non-obese subjects were quantified with independent samples t-test and after bariatric surgery in the obese subjects using repeated-measures *t*-test.

## Results

**Fig. 1:**
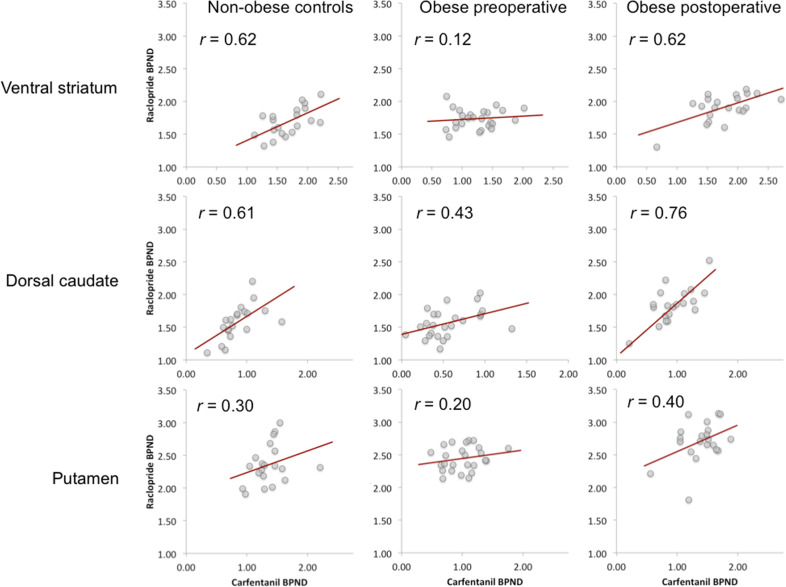
Correlations between [^11^C]raclopride *BP*_ND_ and [^11^C]carfentanil *BP*_ND_ in the ventral striatum, dorsal caudate, and putamen. The association is recovered in the ventral striatum after surgery-induced weight loss. A significant association was found in dorsal caudate in all groups, whereas no association was found in the putamen in any group.

MOR and D_2_R availabilities are presented in the supplementary table (S1). MOR and D_2_R availabilities were associated in the ventral striatum (*r* = .62, *p* < 0.05) and dorsal caudate (*r* = .61, *p* < 0.05) in the control subjects (Fig. 1). Preoperatively, the obese subjects had disrupted association in the ventral striatum (*r* = .12, ns), but the unaltered association in dorsal caudate (*r* = .43, *p* < 0.05) (Fig. 1). MOR and D_2_R availabilities in putamen were not associated in either group.

**Fig. 2:**
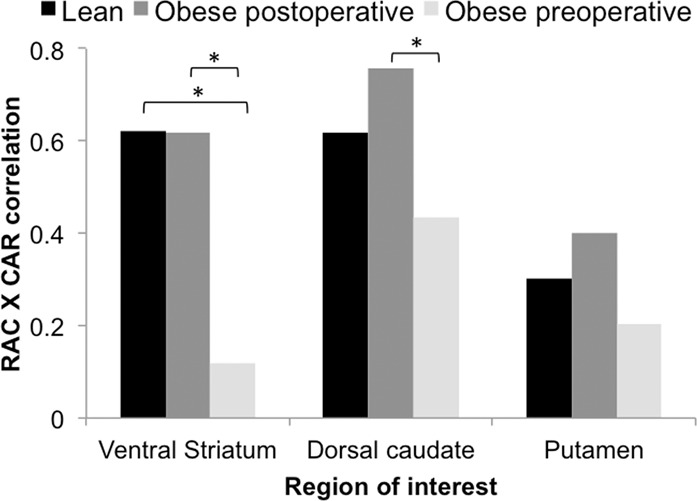
Mean correlations between [^11^C]raclopride *BP*_ND_ and [^11^C]carfentanil *BP*_ND_ in non-obese and obese subjects before and after surgery. Asterisks denote significant between-groups differences.

The association between MOR and D_2_R availabilities in the ventral striatum was recovered (*r* = .62, *p* < 0.05) among obese subjects following the surgery-induced weight loss (mean total weight loss 25.0 ± 8.2 kg and 22.1 ± 6.1%) (Figs. 1 and 2). There was no difference between the two surgical procedures in receptor availabilities or the association between receptors before or after surgery.

In the volumetric analysis of striatal areas, there was no significant difference in striatal volumes between preoperative obese subjects and controls in any ROI (*p*s > 0.05; Table 2). Weight loss did not influence volumes in any region (*p*s > 0.05; Table 2).

**Table 2:** Volumes of striatal areas.

	Obese preoperative (*N* = 25)	*p*^A^	Obese postoperative (*N* = 21)	*p*^B^	Healthy control subjects (*N* = 20)
Ventral striatum (mm^3^)	890 ± 103	0,30	930 ± 131	0,40	925 ± 120
Dorsal caudate (mm^3^)	6623 ± 812	0,99	6634 ± 918	0,20	6319 ± 582
Putamen (mm^3^)	9054 ± 966	0,81	8995 ± 982	0,15	8550 ± 1116

Data are presented as mean ± SD. *P* value columns indicate differences between preoperative and postoperative measurements of the obese subjects (*p*^A^) and between preoperative obese subjects and control subjects (*p*^B^).

## Discussion

Our main finding was that opioid-dopamine interaction is recovered by bariatric surgery and concomitant weight loss. No change in striatal volumes was observed postoperatively. Dysfunctional opioid-dopamine interaction in the ventral striatum is associated with an obese phenotype and may mediate excessive energy uptake, and we have reported earlier that MOR levels return to normal after weight loss^34^. Behaviorally this is in line with previous studies, showing improved satiety and lowered appetite after bariatric surgery^41,42^. We have previously shown that striatal opioid and dopamine systems are coupled in non-obese but not in obese subjects^29^. In the normal-weight subjects, the interaction was strongest in the ventral striatum, but also significant in the dorsal caudate. Growing evidence indicates that MOR and D_2_R are expressed in the same striatal neurons^26,43^. The interaction between these receptor systems is likely crucial in regulating appetite, because it breaks down in the striatum in the obese subjects, while association in the dorsal caudate remains intact. This might explain unaltered D_2_R levels in obesity: although obesity-dependent dysfunction in the dopaminergic system is shown in numerous animal studies, it may be mediated through MOR-dependent mechanisms without having any effect on the actual number of D_2_R proteins. Even if the amount of D_2_R protein stays the same in obesity, decoupling of MOR and D_2_R in the striatum may cause altered dopaminergic functions.

MORs are co-localized with D_2_Rs in striosomes^44^. Dopaminergic neurons in the striosomes project directly to the ventral tegmental area (VTA) and substantia nigra, whereas neurons projecting to GABAergic neurons are distributed in the matrix compartment^45^. Pathways projecting from striosomes back to the midbrain exert disinhibitory control over the dopaminergic neurons^46^, thus having a direct influence on the reward functions. These neurons are under direct opioidergic control^47^. Accordingly, endogenous opioids disinhibit the neurons projecting from the patches to the midbrain (i.e., disinhibiting the disinhibiting neurons), and in this way increase dopaminergic firing in VTA. The rewarding effects of opioids are dependent on the MORs located in the striosomes^48^

Based on the observation that dopamine release caused by opioids in the striatum is dependent on the MORs in the striosomes in mice^48^, we hypothesize that aberrant opioid function in obese humans might lead to diminished dopamine release caused by eating. When obese subjects lose weight, the interaction between MOR and D_2_R is reverted. This further supports the notion that the interaction between these receptor systems is a normal state. The dysfunction of opioid-dopamine interaction in the ventral striatum might be an important factor underlying overeating, and thus a feasible target for pharmacological and behavioral interventions. This has already been noted in pharmacological studies. MOR antagonist naltrexone therapy alone does not lead to significant weight loss, but promising results are obtained when it is coupled with bupropion (a dopamine and norepinephrine reuptake inhibitor)^49–52^. Combination therapy of naltrexone and bupropion has been approved by FDA and EMA for weight management in adults^53^ and a certain amount of obese patients achieve significant weight loss^54–56^. The favorable effect of the combination therapy may be due to the tight coupling of MOR and D_2_R. Moreover, the better efficacy of the combination therapy over monotherapies underlines the complex pattern of neurotransmitter networks underlying overeating and suggests that both aspects of reward functions—*wanting* and *liking*, processes mediated by dopaminergic and opioidergic systems, respectively^57^—have to be taken care of in order to treat obese patients.

This study has certain limitations. Only female subjects were studied, and the results may not be generalizable to male subjects. It was not possible to differentiate the combined effects of postoperative weight loss and altered gut anatomy and function. Altered neuroreceptor interaction may be due to the changes in gut hormones but also due to reduced intake of palatable foods. Further studies are needed to elucidate the sole effect of weight loss due to altered energy intake on the interaction of opioid and dopamine receptors by comparing the effects of weight loss by surgery versus dieting.

## Conclusions

Obesity is associated with disrupted opioid-dopamine interaction in the ventral striatum, but this is recovered by weight loss after bariatric surgery. The dysfunction of opioid-dopamine interaction might be an important factor underlying overeating.

## Supplementary information

S1 Supplementary material
