# Supplementary material for: Mesolimbic opioid-dopamine interaction is disrupted in obesity but recovered by weight loss following bariatric surgery
Source: Transl Psychiatry. 2021 May 1;11:259. doi: 10.1038/s41398-021-01370-2 (PMC8088437; doi:10.1038/s41398-021-01370-2)
Supplement: Supplementary file 1 — S1 Supplementary material [file 41398_2021_1370_MOESM1_ESM.docx]

S1. Binding potentials (as *BP*_ND_) in each ROI for each radiotracer and subject group. Data are presented as mean ± SD.

|  | Obese preoperative | Obese postoperative | Healthy control subjects |
| --- | --- | --- | --- |
| [^11^C]carfentanil (*BP*_ND_) |  |  |  |
| Ventral striatum | 1.457 ± 0.363 | 1.777 ± 0.438 | 1.919 ± 0.366 |
| Dorsal caudate | 0.721 ± 0.331 | 0.901 ± 0.352 | 1.051 ± 0.348 |
| Putamen | 1.099 ± 0.314 | 1.385 ± 0.302 | 1.466 ± 0.308 |
| [^11^C]raclopride (*BP*_ND_) |  |  |  |
| Ventral striatum | 1.913 ± 0.147 | 1.912 ± 0.214 | 1.869 ± 0.216 |
| Dorsal caudate | 1.861 ± 0.240 | 1.787 ± 0.380 | 1.851 ± 0.251 |
| Putamen | 2.758 ± 0.210 | 2.707 ± 0.306 | 2.651 ± 0.328 |
